# Effect of spermidine intake on skeletal muscle regeneration after chemical injury in male mice

**DOI:** 10.14814/phy2.70092

**Published:** 2024-10-24

**Authors:** Tomohiro Iwata, Takanaga Shirai, Kazuki Uemichi, Riku Tanimura, Tohru Takemasa

**Affiliations:** ^1^ Graduate School of Comprehensive Human Sciences University of Tsukuba Tsukuba Ibaraki Japan; ^2^ Japan Society for Promotion Science Chiyoda Tokyo Japan; ^3^ Department of Human Sciences Kanagawa University Yokohama Kanagawa Japan; ^4^ Faculty of Sport and Health Science Ritsumeikan University Kusatsu Shiga Japan; ^5^ Institute of Health and Sport Sciences University of Tsukuba Tsukuba Ibaraki Japan

**Keywords:** autophagy, mitochondria, mTOR signaling, skeletal muscle regeneration, spermidine

## Abstract

Skeletal muscle has a high regenerative ability and maintains homeostasis by rapidly regenerating from frequent damage caused by intense exercise or trauma. In sports, skeletal muscle damage occurs frequently due to intense exercise, so practical methods to promote skeletal muscle regeneration are required. Recent studies have shown that it may be possible to promote skeletal muscle regeneration through new pathways, such as promoting autophagy and improving mitochondrial function. Spermidine is a type of polyamine, and oral intake of spermidine promotes autophagy and improves mitochondrial function without inhibiting mTOR. Therefore, we evaluate the effects of spermidine intake on skeletal muscle regeneration after injury using a mouse model of cardiotoxin‐induced muscle injury. Our results showed no significant change in skeletal muscle wet weight with spermidine intake at all time points. In addition, although spermidine intake significantly increased the mean fiber cross‐sectional area 14 days after injury, these effects were not observed at other time points. In addition, we analyzed stem cells, autophagy, mTOR signaling, inflammation, and mitochondria, but no significant effects of spermidine intake were observed at almost all time points and protein expression levels. Therefore, spermidine intake does not affect skeletal muscle regeneration after chemical injury, and if there is any, it is very limited.

## 1 INTRODUCTION

Skeletal muscle plays an important role in locomotion and metabolism and are essential not only for maintaining and improving health, but also for athletes' performance. Sports activities can sometimes cause skeletal muscle injury (Friden & Lieber, 2001). Skeletal muscles have a strong regenerative capacity and maintain homeostasis by rapidly regenerating from damage caused by intense exercise or trauma. After injury, skeletal muscle stem cells form new myofibers through proliferation, differentiation, and fusion (Dumont et al., 2015). Pax7 is crucial for maintaining and self‐renewing muscle stem cells (satellite cells) (Relaix et al., 2005; Seale et al., 2000). Myf5 marks early myogenic commitment in quiescent satellite cells, while MyoD is key for their activation and differentiation into myoblasts in the early stages of muscle regeneration (Kassar‐Duchossoy et al., 2004; Rudnicki et al., 1992). Myogenin regulates the terminal differentiation of myoblasts into mature myofibers (Nabeshima et al., 1993).

To date, several practical methods have been reported to promote skeletal muscle regeneration. It has been reported that icing promotes recovery from skeletal muscle injury accompanied by small‐scale necrosis (Nagata et al., 2023). It has also been reported that leucine intake promotes skeletal muscle regeneration after cryolesion‐induced muscle injury (Pereira et al., 2014). Recent studies have reported promoting skeletal muscle regeneration through different pathways, which may provide new practical methods for promoting skeletal muscle regeneration. Autophagy is one of the major degradative pathways that plays a role in maintaining the intracellular environment by removing unnecessary proteins and damaged organelles (Parzych & Klionsky, 2014). A recent study reported that activation autophagy through deletion of the ARHGEF3 gene (a RhoA/B‐specific GEF) promote skeletal muscle regeneration (You et al., 2021). On the other hand, mTOR, one of the inhibitory upstream factors of autophagy, is a major growth factor that responds to nutrients and extracellular stimuli (Laplante & Sabatini, 2012). Since mTOR is essential for skeletal muscle regeneration (Ge et al., 2009), it may be effective to promote autophagy without inhibiting mTOR. Mitochondria are intracellular organelles responsible for ATP production by oxidative phosphorylation (Tarasov et al., 2012). Recent studies have shown that transplantation of healthy mitochondria promotes skeletal muscle regeneration (Alway et al., 2023).

Spermidine is a type of polyamine, and its oral intake has been reported to have many beneficial health effects, including cardioprotection, neuroprotection, and life extension (Eisenberg et al., 2016; Madeo et al., 2018). Spermidine has been suggested to activate autophagy without suppressing mTOR signaling (Hofer et al., 2022; Puleston et al., 2014). Spermidine has also been reported to improve mitochondrial function by promoting mitochondrial protein translation and mitophagy (Hofer et al., 2022). In the few studies targeting skeletal muscle have shown that spermidine intake has beneficial effects on skeletal muscle. Spermidine intake has been reported to suppress immobilization‐induced skeletal muscle atrophy (Segales et al., 2020). In addition, the combination of exercise and spermidine intake has been reported to suppress D‐gal‐induced skeletal muscle atrophy, a sarcopenia model (Fan et al., 2017). Furthermore, oral intake and intraperitoneal injection of spermidine have been reported to reduce muscle damage in collagen VI‐null mice (Chrisam et al., 2015).

However, no studies have examined the effects of spermidine intake on muscle regeneration after injury. Therefore, we hypothesized that spermidine intake promotes muscle regeneration after injury by promoting autophagy without mTOR suppression and improving mitochondrial function. Therefore, we evaluated the effects of spermidine intake on skeletal muscle regeneration after injury using a mouse model of cardiotoxin‐induced muscle injury.

## 2 MATERIALS AND METHODS

### 2.1 Animals

All experimental procedures performed in this study wereapproved by the Institutional Animal Experiment Committeeof the University of Tsukuba (animal ethical approval number23‐380) based on the guidelines of the National Institutes ofHealth for the Care and Use of Laboratory Animals (NationalResearch Council Committee for the Update of the Guide forthe and Use of Laboratory 2011). Male 7‐week‐old C57BL/6j mice (The Jackson Laboratory Japan, Inc., Kanagawa, Japan) were used in this study. The mice were kept in temperature (22 ± 2°C) and humidity (55% ± 5%) controlled facilities under a 12/12 h light/dark cycle with ad libitum access to food (23.1% protein:5.1% fat:71.8% carbohydrate; Oriental, Japan) and distilled water. After 1 week of acclimatization, 50 μL of 10 μM cardiotoxin (CTX) (L8102; LAT) in PBS was injected into the tibialis anterior muscle of both legs of each mouse (Guardiola et al., 2017; Hayashi et al., 2023). Grouping was performed immediately after the injection of CTX. Mice were randomly assigned to groups. The DW group had ad libitum access to distilled water, and the SP group had ad libitum access to a 5 mM concentration of spermidine (14,918; CAY) mixed in distilled water (Ito et al., 2021). As a normal control, the group had ad libitum access to distilled water without injecting CTX (*n* = 6 per group). Mice were euthanized under anesthesia at 4‐, 7‐, 14‐, and 21 days post‐injury. After measuring the body weight, the muscles were removed, immediately frozen in liquid nitrogen, and stored at −80°C until further analysis.

### 2.2 Western blotting

Dissected tibialis anterior muscles were immediately frozen in liquid nitrogen, and total muscle protein was extracted using a homogenizing solution containing 50 mM HEPES (pH 7.6), 150 mM NaCl, 10 mM EDTA, 10 mM Na_4_P_2_O_7_, 10 mM NaF, 2 mM Na_3_VO_4_, 1% (v/v) NP‐40, 1% (v/v) Na‐deoxycholate, 0.2% (w/v) SDS, and 1% (v/v) complete protease inhibitor cocktail (169‐26, 063; Wako). The protein concentrations were measured using a Protein Assay Bicinchoninate Kit (297‐73, 101; Wako). Before SDS‐PAGE separation, an aliquot of the extracted protein solution was combined with an equal volume of sample loading buffer containing 1% (v/v) 2‐mercaptoethanol, 4% (w/v) SDS, 125 mM Tris–HCl (pH: 6.8), 10% (w/v) sucrose, and 0.01% (w/v) bromophenol blue. The mixture was heated at 37°C for 1 h. Ten micrograms of each protein sample were separated on an SDS‐PAGE and electrically transferred to an ImmunoBlot PVDF membrane (#1620177; Bio‐Rad Laboratories). Blots were blocked for 1 h at room temperature with a blocking solution (5% skim milk (Yukijirushi), 5% Blocking One (03953‐95; Nacalai Tesque Inc.), and 0.1% Tween‐20 in TBS) with primary antibodies in TBS containing 0.1% Tween‐20 and incubated overnight at 4°C. After incubation, the membranes were incubated with a horseradish peroxidase‐conjugated secondary antibody Anti rabbit IgG (7074P2; CST), Anti mouse IgG (7076P2; CST) or Anti goat IgG (HAF017; R&D Systems) for 60 min at room temperature. The signals were detected using Immunostar Zeta or LD (295‐72, 404/299‐69, 904; Wako Chemicals, Osaka, Japan), quantified with C‐Digit (LI‐COR Biosciences, Lincoln, NE, USA), and expressed in arbitrary units. Coomassie brilliant blue staining was used to verify consistent loading.

### 2.3 Primary antibodies for western blotting

The following primary antibodies were used for western blotting: anti‐Pax‐7 (sc‐81,648; Santa Cruz), anti‐Myf5 (C‐20, sc‐302; Santa Cruz), anti‐MyoD (5.8A, sc‐32,758; Santa Cruz), anti‐myogenin (sc‐12,737; Santa Cruz), anti‐MAP1LC3 Microtubule‐associated protein 1 light chain 3 (LC3) (4108; CST), anti‐p62 (SQSTM1) (PM045; MBL), anti‐ubiquitin (sc‐166,553; Santa Cruz), anti‐eIF4E (C46H6, #2067; CST), anti‐p‐eIF4E (S209, #9741; CST), anti‐rpS6 (#2217; CST), anti‐p‐rpS6 (Ser240/244, #5364P; CST), anti‐p‐rpS6 (Ser235/236; #4858S, CST), anti‐oxidative phosphorylation (OXPHOS) (ab110413; abcam), anti‐cytochrome c (ab13575; abcam), anti‐PGC‐1α (516,557; Millipore), anti‐TNF‐α (TN3‐19, 12) (sc‐12,744; Santa Cruz), anti‐IL‐6 (AF406NA; R&D Systems), and anti‐IL‐1β (H153, sc‐7884; Santa Cruz).

### 2.4 Immunohistochemistry and cross‐sectional area analysis

The tibialis anterior muscles were covered with an optimal cutting temperature compound (4583; Sakura Finetek, Tokyo, Japan), frozen in liquid nitrogen‐cooled isopentane, and stored at −20°C until sectioning. Frozen tibialis anterior muscles were sliced into 10‐μm sections using a cryostat (NX70; ThermoFisher Scientific K.K., Tokyo, Japan). The sections were fixed in 4% paraformaldehyde, permeabilized with 0.1% Triton X‐100, and blocked with M.O.M. blocking reagent (MKB‐2213; Vector Laboratories, Newark, CA, USA). For PAX7 staining, the sections underwent antigen retrieval with DAKO buffer (S1699; Agilent, Santa Clara, CA, USA) for 10 min at 110°C, followed by blocking (Hayashi et al., 2023). All sections were incubated with primary antibodies overnight at 4°C, and then incubated with secondary antibodies for 1 h at room temperature in the dark. For cross‐sectional area (CSA) analysis, the antibodies were diluted with 0.1% TritonX‐100, 1% horse serum (12,449; JRH biosciences) in PBS, and the primary antibody was Rat anti‐Laminin α‐2 (4H8‐2; sc‐59,854; Santa Cruz), and the secondary antibody was Goat anti‐rat IgG Alexa Fluor‐546 (A11035; Invitrogen). After incubation with DAPI (62,248; Invitrogen) for 1 min, the sections were mounted with Fluorescent Mounting Medium (5570–0005; KPL). For skeletal muscle stem cell analysis, antibodies were diluted with 1/30 M.O.M. protein concentrate (BMK‐2202; Vector Laboratories) in PBS, and mouse anti‐PAX7 (AB_528428; Developmental Studies Hybridoma Bank (DSHB)), rabbit anti‐MyoD (AB133627; abcam), and rat anti‐LAMININ (4H8‐2; sc‐59,854; Santa Cruz) were used as primary antibodies, and goat anti‐mouse IgG1 Alexa Fluor‐488 (A21121; Invitrogen), goat anti‐rabbit IgG Alexa Fluor Plus‐555 (A32732; Invitrogen) and donkey anti‐rat IgG Alexa Fluor‐647 (Ab150155; abcam) were used as secondary antibodies. The sections were mounted with VECTASHIELD Vibrance Antifade Mounting Medium with DAPI (H‐1800; Vector Laboratories; USA). The cross‐sectional area (CSA) of the myofiber sections was measured using laminin‐α2‐stained images at 10× magnification. Images were acquired using an all‐in‐one fluorescence microscope BZ‐X710, with a CFI Plan Fluor 10×/20× objective lens. (Keyence, Osaka, Japan). The deep learning algorithm CellPose was used to automatically segment the individual fibers and extract their contours (Stringer et al., 2021). Based on the extracted contours, the myofiber CSA was quantified using FIJI/ImageJ (Schindelin et al., 2012). Because almost all myofibers had a central nucleus, which is a characteristic of regenerated muscle, we analyzed skeletal muscle stem cells and myofiber CSA in whole muscles.

### 2.5 Statistical analysis

All data are expressed as the mean ± standard error (SE). A two‐tailed unpaired *t*‐test was performed. Statistical significance was defined as *p* < 0.05 (Prism 5.0; GraphPad, La Jolla, CA), and GraphPad Prism 8 (GraphPad, Inc.) was used for all statistical calculations.

## 3 RESULTS

### 3.1 Body weight, skeletal muscle wet weight, and myofiber cross‐sectional area

**FIGURE 1:**
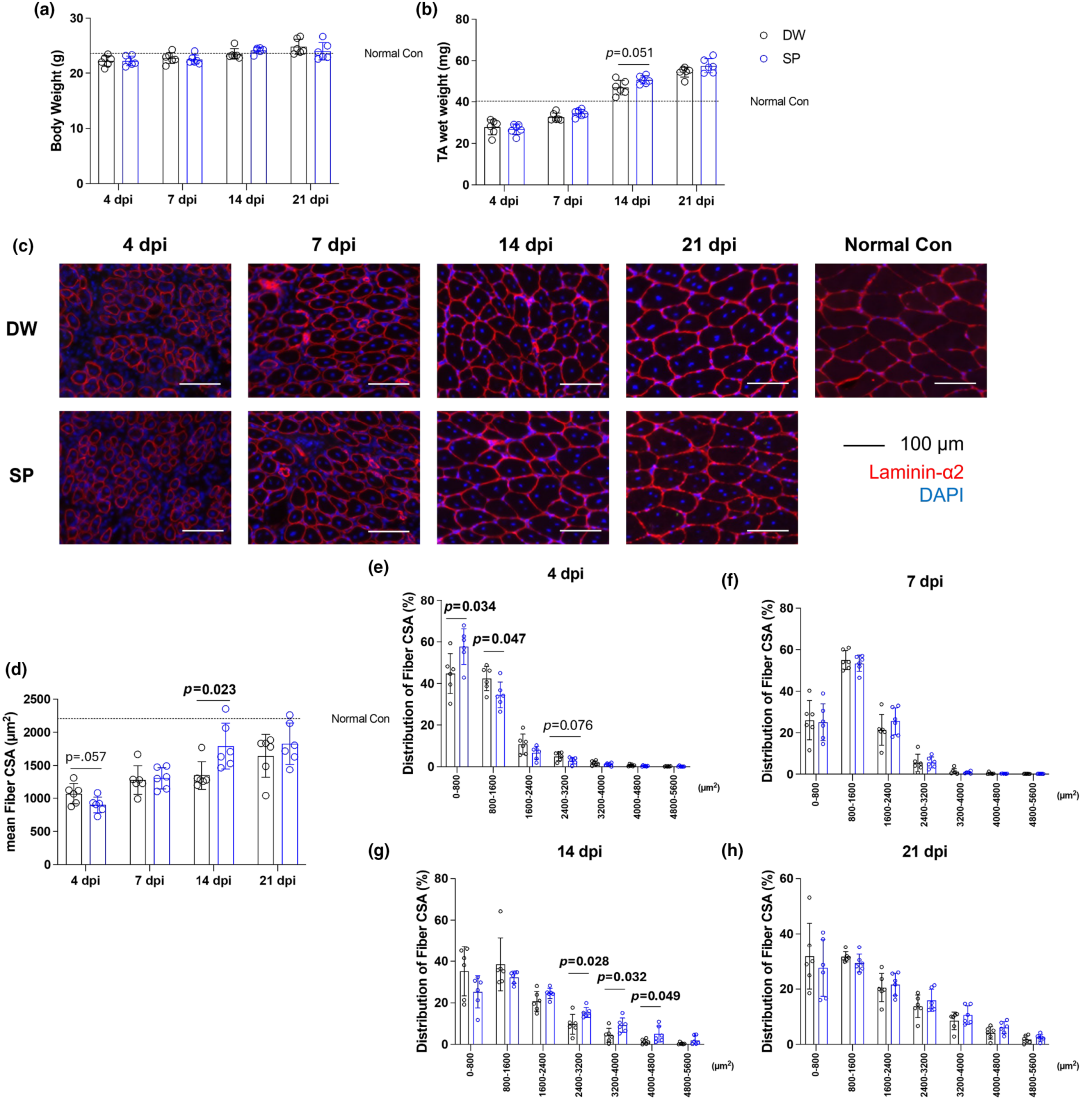
Body weight, muscle wet weight, and myofiber cross‐sectional area (CSA). (a) Body weight; (b) TA wet weight; (c) representative immunohistochemistry images; (d) mean fiber CSA; (e) fiber distribution 4 days post‐injury; (f) fiber distribution 7 days post‐injury; (g) fiber distribution 14 days post‐injury; and (h) fiber distribution 21 days post‐injury. The data are present in each plot as the mean ± standard deviation (*n* = 6 per group). dpi, days post‐injury; DW, distilled water group; SP, spermidine group.

We evaluated the effects of spermidine intake on body weight, skeletal muscle wet weight, and mean myofiber CSA after CTX‐induced skeletal muscle injury (Figure 1). Spermidine intake had no significant effect on body weight and CSA at any time point (Figure 1a,b). However, at 14 days post‐injury, spermidine intake significantly increased myofiber mean CSA was (*p* = 0.022) (Figure 1c,d). The frequency distribution of myofiber CSA at each time point is shown Figure 1e–h. At 4 days post‐injury, spermidine intake significantly increased the frequency of myofibers between 0 and 800 μm^2^ (*p* = 0.034), significantly decreased the frequency of myofibers between 800 and 1600 μm^2^ (*p* = 0.047) (Figure 1e). At 14 days post‐injury, spermidine intake significantly increased the frequency of myofibers of 2400–3200 μm^2^ (*p* = 0.028), 3200–4000 μm^2^ (*p* = 0.032), and 4000–4800 μm^2^ (*p* = 0.049) (Figure 1g).

### 3.2 Skeletal muscle stem cells

**FIGURE 2:**
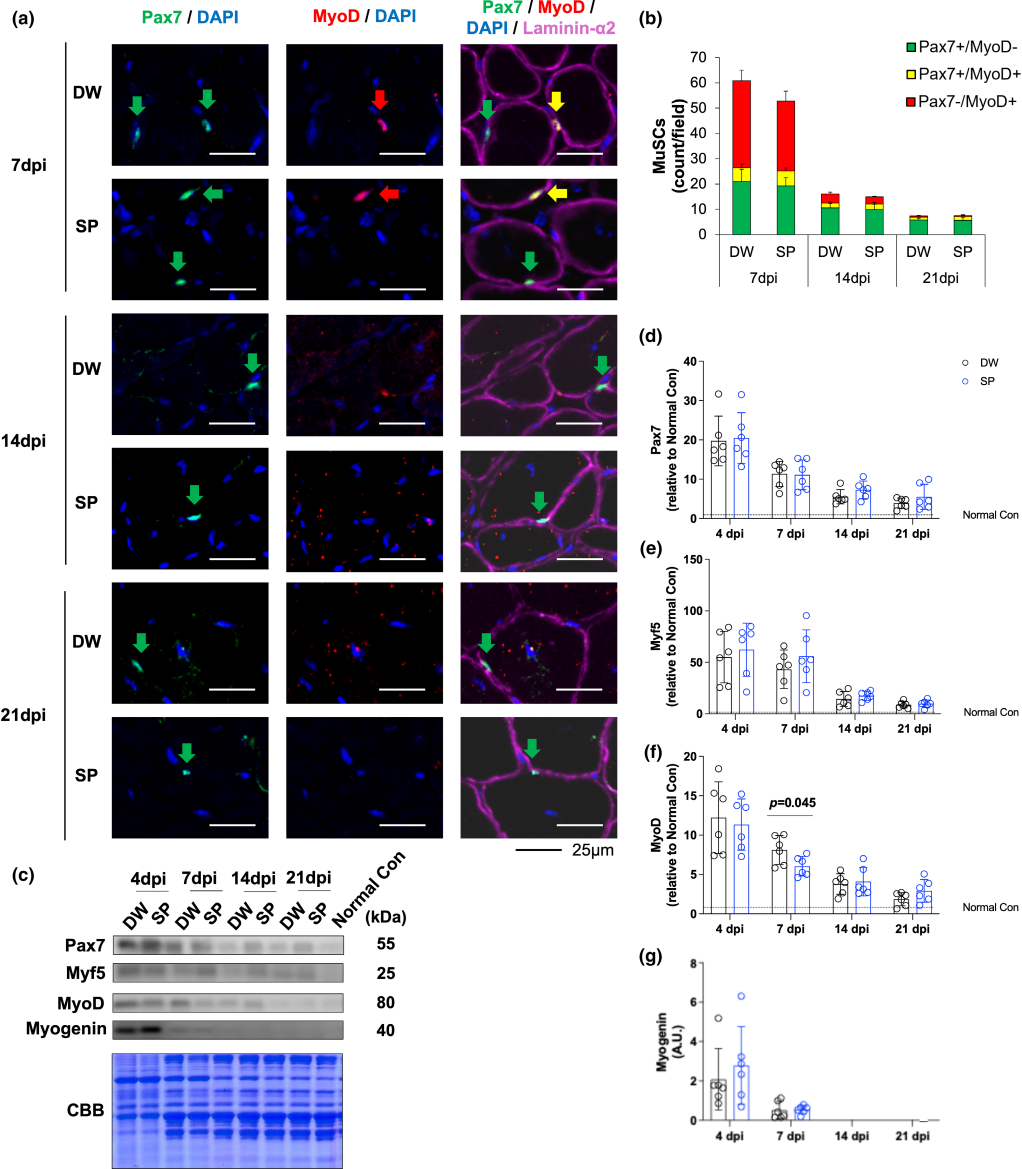
Muscle stem cells. (a) Representative immunohistochemistry images; (b) proportion of muscle stem cells (MuSCs); (c) Representative band image and Coomassie brilliant blue staining of the total amount of protein; (d) Pax7; (e) Myf5; (f) MyoD; and (g) Myogenin. The data are present in each plot as the mean ± standard deviation (*n* = 6 per group). dpi, days post‐injury; DW, distilled water group; SP, spermidine group.

To evaluate the effect of spermidine intake on skeletal muscle stem cells after CTX‐induced skeletal muscle injury, immunofluorescence staining was performed (Figure 2). Pax7 and MyoD were quantified by immunofluorescence staining, and the proportion of Pax7+/MyoD−, Pax7+/MyoD+, and Pax7−/MyoD+ cells is shown. Data at 4 days post‐injury are not shown because there was excessive nuclear infiltration and unstable cell membranes, and it was challenging to exclude non‐specific fluorescence based on the overlap between the nucleus and cell membrane. However, at 7‐, 14‐, and 21 days post‐injury, spermidine intake did not significantly alter count of Pax7+/MyoD−, Pax7+/MyoD+, and Pax7−/MyoD+ nucleis (Figure 2a,b). Western blotting was used to evaluate the expression levels Pax7, Myf5, MyoD, and Myogenin (Figure 2c–g). At 7 days post‐injury, spermidine intake significantly reduced MyoD protein expression levels (*p* = 0.022) (Figure 2c,f). However, spermidine intake had no significant effect on all other proteins and time points examined (Figure 2c–e,g).

### 3.3 Autophagy‐related proteins

**FIGURE 3:**
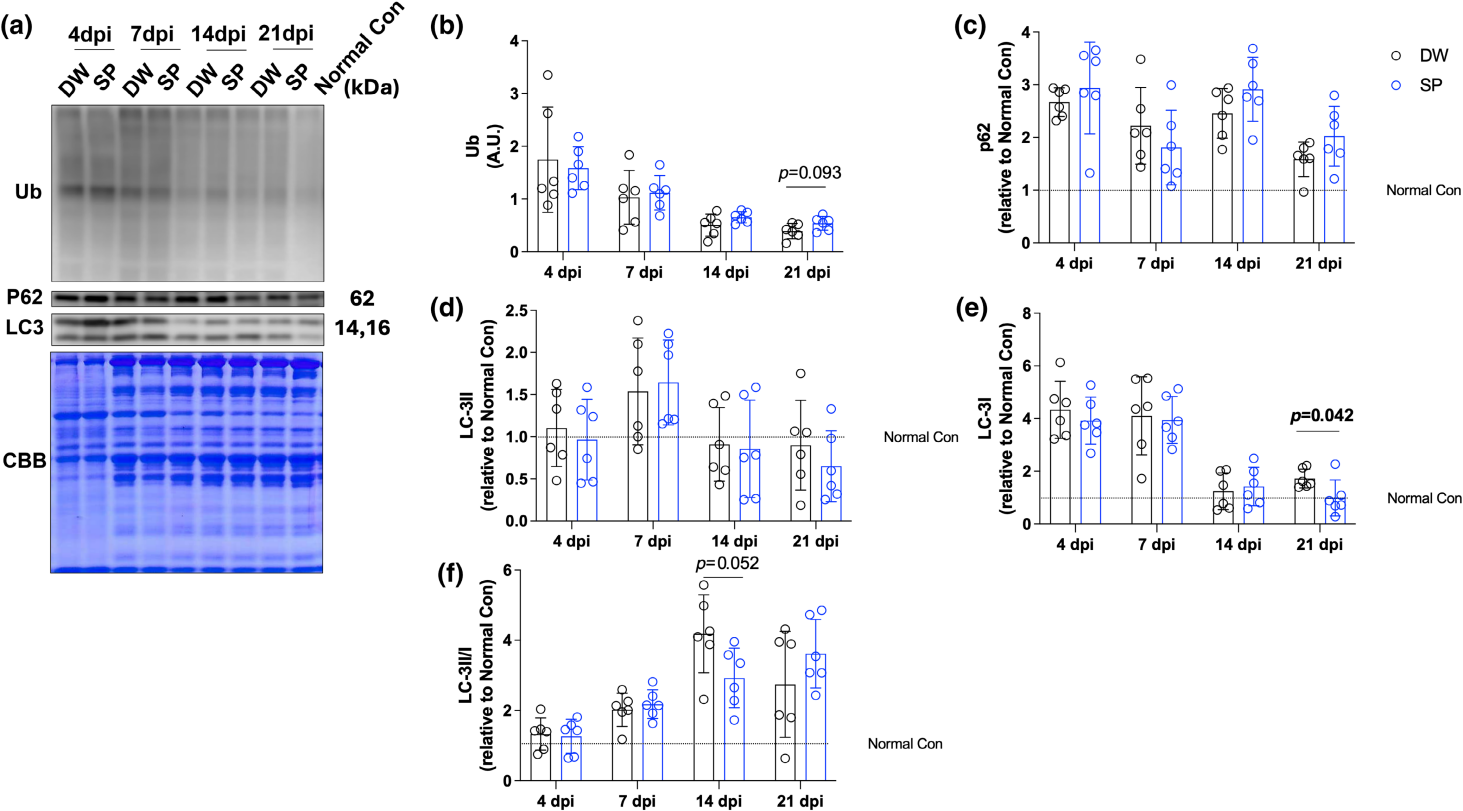
Autophagy‐related proteins. (a) Representative band image and Coomassie brilliant blue staining of the total amount of protein; (b) ubiquitinated proteins; (c) p62; (d) LC3‐II; (e) LC3‐I; and (f) LC3‐II/I. The data are present in each plot as the mean ± standard deviation (*n* = 6 per group). dpi, days post‐injury; DW, distilled water group; SP, spermidine group.

To evaluate the effect of spermidine intake on autophagy after CTX‐induced skeletal muscle injury, autophagy‐related proteins were analyzed by western blotting (Figure 3). We evaluated the protein expression levels of LC3 I and II (markers for phagophores and autophagosomes, respectively), ubiquitinated proteins (damaged proteins), and P62 (autophagy substrates). At 21 days post‐injury, spermidine intake significantly decreased the protein expression level of LC3‐I (Figure 3a,e; *p* = 0.042, respectively). However, spermidine intake had no significant effect on all other proteins and time points examined (Figure 3a–d,f).

### 3.4 mTOR signaling

**FIGURE 4:**
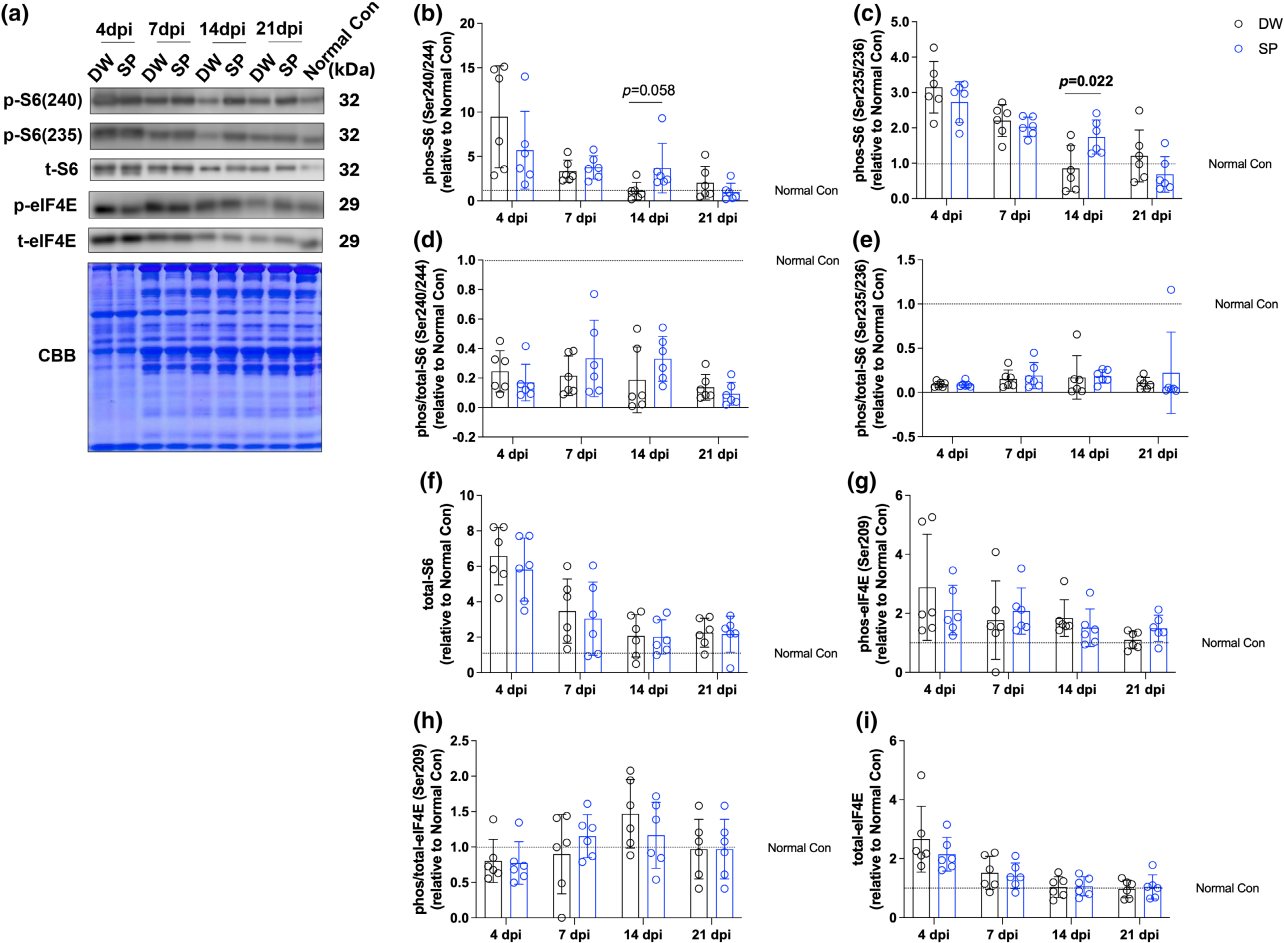
mTOR signaling. (a) Representative band image and Coomassie brilliant blue staining of the total amount of protein; (b) p‐S6 (Ser240/244); (c) p‐S6 (Ser235/236); (d) p/t‐S6 (Ser240/244); (e) p/t‐S6 (Ser235/236); (f) t‐S6; (g) p‐eIF4E(Ser209); (h) p/t‐eIF4E(Ser209); and (i) t‐eIF4E. The data are present in each plot as the mean ± standard deviation (*n* = 6 per group). dpi, days post‐injury; DW, distilled water group; SP, spermidine group.

To evaluate the effect of spermidine intake on mTOR signaling after CTX‐induced skeletal muscle injury, we analyzed the phosphorylation level of S6 and eIF4E the downstream protein of mTOR signaling, by western blotting (Figure 4). At 14 days post‐injury, spermidine intake significantly increased the phosphorylation level of S6 Ser235/236 (*p* = 0.022) (Figure 4a–c). However, spermidine intake had no significant effect on all other proteins and time points examined (Figure 4a,d–i).

### 3.5 Mitochondria‐related proteins

**FIGURE 5:**
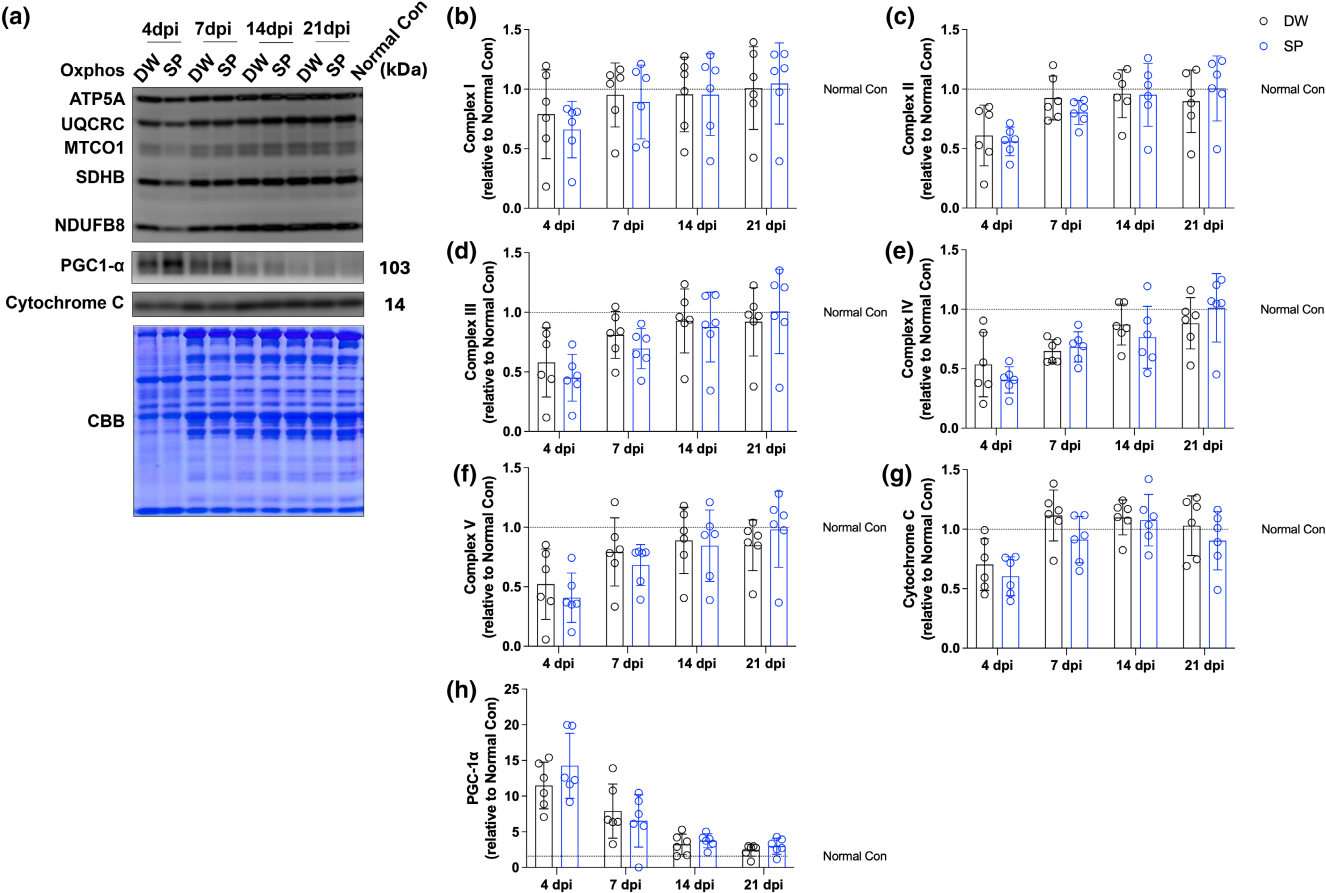
Mitochondria‐related proteins. (a) Representative band image and Coomassie brilliant blue staining of the total amount of protein; (b) Complex I; (c) Complex II; (d) Complex III; (e) Complex IV; (f) Complex V; (g) Cytochrome C; and (h) PGC‐1α. The data are present in each plot as the mean ± standard deviation (*n* = 6 per group). dpi, days post‐injury; DW, distilled water group; SP, spermidine group.

To evaluate the effects of spermidine intake on mitochondria after CTX‐induced skeletal muscle injury, mitochondria‐related proteins were analyzed by western blotting (Figure 5). We analyzed the protein expression levels of Oxphos (NDUFB8, SDHB, UQCRC, MTCO1, ATP5A), Cytochrome C (a protein responsible for ATP production through mitochondrial oxidative phosphorylation), and PGC‐1α (the master regulator of mitochondrial biogenesis). Spermidine intake had no significant effect on all proteins and time points examined (Figure 5a–h).

### 3.6 Inflammatory cytokines

**FIGURE 6:**
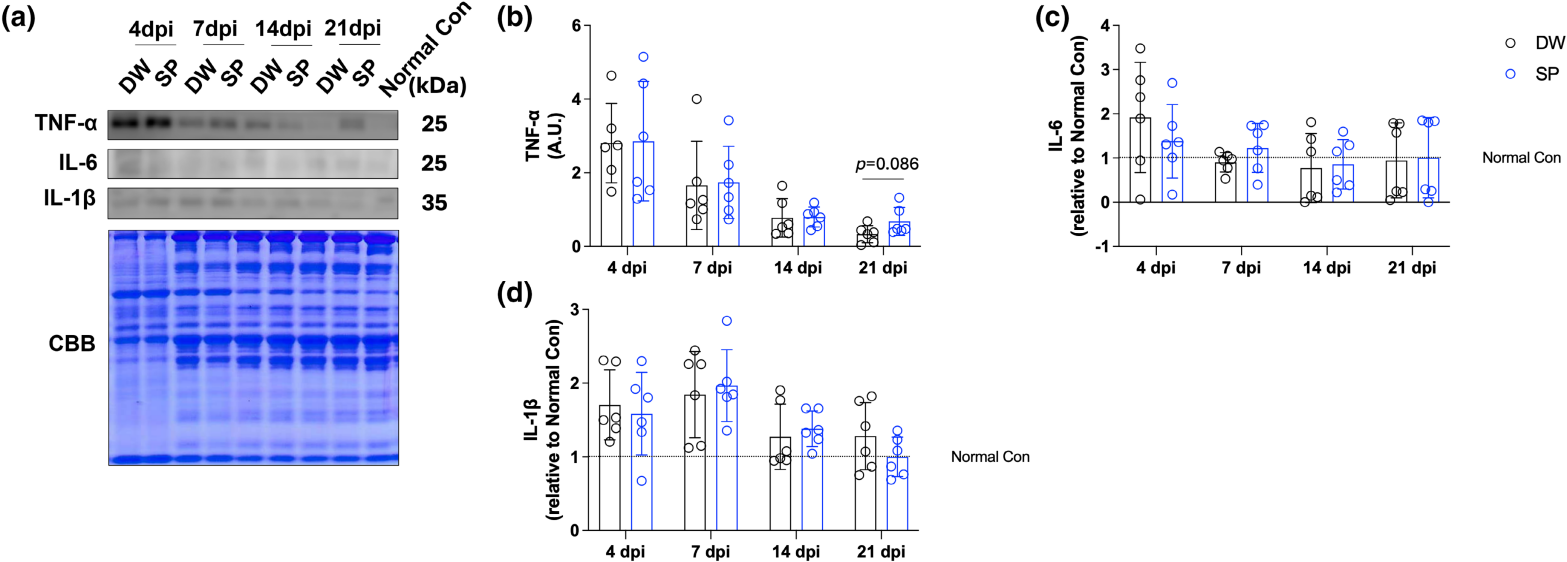
Inflammatory cytokines. (a) Representative band image and Coomassie brilliant blue staining of the total amount of protein; (b) TNF‐α; (c) IL‐6; and (d) IL‐1β. The data are present in each plot as the mean ± standard deviation (*n* = 6 per group). dpi, days post‐injury; DW, distilled water group; SP, spermidine group.

To evaluate the effect of spermidine intake on inflammation after CTX‐induced skeletal muscle injury, inflammatory cytokines were analyzed by western blotting (Figure 6). We evaluated the protein expression of TNF‐α, IL‐6, and IL‐1β. Spermidine intake had no significant effect on all proteins and time points examined (Figure 6a,e).

## 4 DISCUSSION

Our results showed no significant change in skeletal muscle wet weight with spermidine intake at all time points. In addition, although spermidine intake significantly increased the mean fiber CSA 14 days after injury, these effects were not observed at other time points. In addition, we analyzed stem cells, autophagy, mTOR signaling, inflammation, and mitochondria, but no significant effects of spermidine intake were observed at almost all time points and protein expression levels. Therefore, spermidine intake does not affect skeletal muscle regeneration after chemical injury, and if there is any, it is very limited.

Although our results showed that spermidine intake did not affect most of the measurements at all time points, spermidine intake non‐significantly increased skeletal muscle wet weight and significantly increased mean myofiber CSA at Day 14 after injury. Our results also showed that spermidine intake significantly decreased MyoD‐positive nuclei and MyoD protein expression levels at Day 7 after injury. These results suggest that spermidine intake may accelerate the transition of stem cells to a late differentiation stage at Day 7 after injury and promote skeletal muscle regeneration phenotypically at Day 14 after injury. Recent studies have demonstrated that spermidine and its downstream eIF5A are pharmacological targets for activating skeletal muscle stem cells (Zhang et al., 2024). Our results showed no significant changes in protein expression levels other than MyoD, which is noteworthy, although limited. Furthermore, our results demonstrate that spermidine supplementation non‐significantly increased phosphorylation of Ser240/244 and significantly increased phosphorylation of Ser235/236 downstream of mTOR S6 at 14 days post‐injury, when skeletal muscle wet weight was non‐significantly increased and mean myofiber CSA was significantly increased. Our results also demonstrate that spermidine administration non‐significantly decreased LC3‐II/I, an indicator of autophagy activation, at 14 days post‐injury. Phenotypic changes due to spermidine supplementation were observed at 14 days post‐injury, but not at 7 and 21 days post‐injury. The time‐dependent result of increased CSA at 14 days despite no change in CSA at 7 and 21 days post‐injury is similar to a previous study reporting enhanced skeletal muscle regeneration by mitochondrial transplantation (Alway et al., 2023). In addition, some protein expression results support the activation of mTOR signaling, suggesting that spermidine administration may have acted effectively during the anabolic growth period of differentiated muscle fibers. These results also suggest that spermidine intake may activate mTOR signaling at 14 days after injury, thereby suppressing downstream autophagy and increasing skeletal muscle wet weight and CSA. However, it should be noted that these are only predictions from the few measurements that changed. Future studies should actually examine the effect of spermidine on the anabolic growth of differentiated muscle cells using muscle hypertrophy models.

In this study, spermidine intake did not significantly affect autophagy, mitochondria, or inflammation at almost all proteins and time points. Previous studies on skeletal muscle have reported that spermidine administration improved pathologies involving autophagy impairment, mitochondrial damage, and inflammation (Fan et al., 2017; Segales et al., 2020). However, the effect of spermidine intake on healthy muscle has not been examined. Therefore, the effects of spermidine on autophagy, mitochondria, and inflammation in skeletal muscle may be secondary and limited.

In this study, muscle strength, total muscle cross‐sectional area, fiber number, and fibrosis were not examined. Therefore, the changes in muscle fiber CSA observed in regeneration without changes in skeletal muscle wet weight due to spermidine administration are difficult to interpret. The changes in CSA may reflect changes in the relative frequency of muscle fiber types. Future studies should conduct more detailed analyses including the above‐mentioned contents. In addition, future studies should investigate the effect of spermidine on mitochondria in muscle regeneration in detail by analyzing protein synthesis using the SunSET method and autophagy flux assay and mitochondrial quality (Goodman & Hornberger, 2013; Kaizuka et al., 2016; Redmann et al., 2018; Schmidt et al., 2009; Ueno & Komatsu, 2020). Furthermore, this study did not include a group of healthy mice that only received spermidine intake. Future studies should examine the effects of spermidine administration on autophagy and mitochondria in healthy mice. The cardiotoxin injection‐induced skeletal muscle injury model used in this study produces muscle damage that is much more severe than that experienced during sports (Wang et al., 2022). When applying our results for sports, it is necessary to confirm its effectiveness through more practical experiments.

In conclusion, in this study, spermidine intake did not significantly affect the phenotype or levels of almost all proteins related to autophagy, mitochondria, and inflammation at almost all time points in skeletal muscle regeneration after chemical injury.

## AUTHOR CONTRIBUTIONS

**Tomohiro Iwata:** Conceptualization, Investigation, Validation, Data curation, Writing – original draft, artwork, all authors approved the final version of the manuscript. **Takanaga Shirai:** Investigation, Data curation, all authors approved the final version of the manuscript. **Kazuki Uemichi:** Investigation, all authors approved the final version of the manuscript. **Riku Tanimura:** Investigation, all authors approved the final version of the manuscript. **Tohru Takemasa:** Supervision, review & editing, all authors approved the final version of the manuscript.

## FUNDING INFORMATION

This work was supported by JSPS KAKENHI Grant Number 23H03266, 23KJ2060, Leave a Nest Co., Ltd. incu·be award, and IKEDA SCIENTIFIC Co., Ltd. bridge fellowship.

## CONFLICT OF INTEREST STATEMENT

The authors declare that they have no known competing financial interests or personal relationships that could appear to have influenced the work reported in this article.
